# Persistent poverty and breast cancer incidence by tumor subtype: intersections of rural/urban residence and race within USA Surveillance Epidemiology and End Results Registries, 2017 to 2021

**DOI:** 10.1007/s10552-025-02114-z

**Published:** 2026-01-17

**Authors:** Heather R. Sherr, Amr S. Soliman, Kimberly A. Bertrand, Kelly A. Hirko

**Affiliations:** 1 Washington University School of Medicine, St. Louis, MO USA; 2 City University of New York Medical School, New York, NY USA; 3 Slone Epidemiology Center at Boston University, Boston, MA USA; 4 Department of Epidemiology & Biostatistics, College of Human Medicine, Michigan State University, East Lansing, MI USA

**Keywords:** Persistent poverty, Rural, Breast cancer, Subtypes

## Abstract

**Purpose:**

Breast cancer characteristics and outcomes vary by tumor subtype, poverty, race, and geography. Persistent poverty (> 20% residents in poverty for 30+ years) has been associated with breast cancer risk, but whether associations differ by subtype is unknown. We examined subtype-specific breast cancer incidence by persistent poverty, stratified by rurality and race.

**Methods:**

Using county-level Surveillance, Epidemiology, and End Results data from 2017 to 2021 (excluding 2020), we calculated Luminal A, Luminal B, HER2-enriched, and triple-negative breast cancer (TNBC) incidence rates. We estimated rate differences (RDs) by persistent poverty using age-adjusted and multivariable linear regression models, with stratification by rurality and county racial composition.

**Results:**

In age-adjusted models, persistent poverty counties had lower incidence of Luminal A (RD − 13.58, 95% CI = − 19.8, − 7.4) and higher TNBC (RD = 3.82, 95% CI = 2.0, 5.6) compared to non-persistently poor counties. Differences were not significant in multivariable models. In stratified analysis, higher TNBC rates were observed in persistently poor rural (multivariable RD =1.70, 95% CI = 0.3, 4.8) but not urban (multivariable RD = − 1.07, 95% CI = − 4.7, 2.5; *p*_int_ = 0.09) areas. In counties with > 5% non-Hispanic Black population, Luminal B rates were lower in persistently poor vs. non-persistently poor counties (multivariable RD = − 3.96, 95% CI = − 6.7, − 1.2; *p*_int_ = 0.03).

**Conclusion:**

Results from this study suggest that differences in breast cancer subtypes by persistent poverty status are largely explained by other measures of more recent disadvantage including recent poverty, unemployment, uninsurance, and race. Targeted strategies are needed to address breast cancer disparities within socioeconomically disadvantaged communities.

**Supplementary Information:**

The online version contains supplementary material available at 10.1007/s10552-025-02114-z.

## Introduction

Breast cancer incidence rates have been rising by 1% per year since 2012. This increase is largely driven by the detection of early-stage and hormone receptor-positive (HR+) disease [1]. HR+breast cancers, including Luminal A (HR+/Human Epidermal Growth Factors Receptor 2 (HER2)−) and Luminal B (HR+/HER2+) tumor subtypes, have receptors for either estrogen or progesterone, or both, and therefore, are more likely to respond to hormone therapy than other subtypes [2]. The Luminal subtypes represent 80% of all breast cancer diagnoses and have the highest survival rates [3].

Triple-negative breast cancer (TNBC; HR−/HER2−), on the other hand, is unresponsive to hormone therapy and monoclonal antibody treatments, and has the lowest survival rate among all subtypes [3]. TNBC is highly aggressive and is often diagnosed at regional (stage III) and distant (stage IV) stages. Notably, both TNBC and distant-staged breast cancers are more commonly diagnosed in non-Hispanic Black (NHB) and Hispanic women than in non-Hispanic white women [1], highlighting the importance of screening and early diagnosis to improve outcomes in these populations. There are both regional and racial components to TNBC disparities, as TNBC occurs most often in young non-Hispanic Black women and in Southern states, including Mississippi and Louisiana [1, 4].

The aforementioned regions with higher TNBC burden overlap with areas of high poverty, which has been associated with higher prevalence of hormone-negative breast cancers, although results from prior studies are inconsistent [5, 6]. Recent studies, including our own, suggest that persistent poverty is positively associated with late-stage (stage III and IV) breast cancer diagnosis [7, 8]. Persistent poverty may be associated with breast cancer risk over and above current poverty, in part through chronic stress-induced apoptosis inhibition and weakening of immune function [9, 10]. Indeed, prolonged psychosocial stress associated with persistent socioeconomic hardship elevates stress hormones that suppress apoptosis and impair immune surveillance, allowing mutation-prone cells to persist. These stress-related disruptions create a microenvironment conducive to the development of aggressive TNBC, providing a plausible mechanistic link between persistent poverty and elevated TNBC risk [11, 12].

To our knowledge, no studies have assessed the independent association between persistent poverty and breast cancer incidence by tumor subtype. In our prior study, persistently poor counties with small NHB populations had higher rates of late-stage breast cancer diagnosis compared to non-persistently poor counties, while rates of late-stage breast cancer did not differ by persistent poverty in counties with larger NHB populations [8]. We hypothesized that these findings may be explained by the higher prevalence of TNBC in both impoverished and non-impoverished Black populations, warranting additional analysis to elucidate breast cancer subtype distributions according to persistent poverty. This study extends our previous work by assessing whether persistent poverty is associated with breast cancer subtypes after accounting for more recently experienced poverty. Since many persistently poor counties are rural and have larger Non-Hispanic Black populations, we also evaluated associations between persistent poverty and breast cancer tumor subtype according to county-level rural/urban status and proportion of NHB residents.

## Methods

### Study population

Breast cancer data are sourced from the National Cancer Institute’s Surveillance, Epidemiology, and End Results (SEER) Research Plus Limited-Field 22 database, April 2024 release, including data from 22 cancer registries [13]. SEER includes nationally representative data on cancer diagnoses, treatment, and survival.

Of the 1,086 counties included in the SEER Research Plus Limited-Field 22 database, our study included breast cancer incidence data from 1,079 counties. The Alaska Native Registry and six additional counties (Clark County, Idaho, Borden County, Texas, Glasscock County, Texas, Kenedy County, Texas, King County, Texas, and Loving County, Texas) were excluded from the study, as they lacked information on persistent poverty status. Breast cancer cases were defined using the ICD-O-3/WHO 2008 site recode included in SEER [14]. We assessed the most recent five-year period of breast cancer incidence rates at the time of analysis (2017–2021). All diagnoses from the year 2020 were excluded due to data quality concerns related to incidence reporting during the first year of the COVID-19 pandemic, as well as delayed mammography screenings [15, 16]. Cancer cases were restricted to those diagnosed above the age of 20.

### Primary exposure

Persistent poverty is defined by the United States Department of Agriculture (USDA) as counties with 20% or more of the population living below the poverty line for about 30 years [17]. This dichotomous measure is derived from the 1990 and 2000 decennial censuses, as well as the 2007–2011 and 2015–2019 American Community Survey (ACS) 5-year estimates.

### Primary outcome

The primary outcome for this study was the county-level age-adjusted breast cancer incidence rates per 100,000 person-years from 2017 to 2021 (excluding 2020). We focused on the first primary breast cancer diagnoses, selecting only the first matching record to exclude recurrent and secondary diagnoses. Our outcome of breast cancer differentiated by subtype using the Breast Subtype (2010+) variable in SEER*Stat. This variable combines information from the estrogen receptor (ER) Status Recode Breast Cancer (1990+), progesterone receptor (PR) Status Recode Breast Cancer (1990+), and Derived HER2 Recode (2010+) [18]. Categorizations are derived from clinical test results of HER2, ER, and PR markers recorded in patient medical records. Tumor subtypes were classified as Luminal A (ER-positive and/or PR-positive and HER2-negative with grade 1 or 2); Luminal B (either ER-positive and/or PR-positive and HER2-positive or ER-positive and/or PR-positive and HER2-negative with grade 3); HER2-enriched (ER-negative, PR-negative, and HER2-positive); TNBC (ER-negative, PR-negative, and HER2-negative) [19]. Given that not all breast tumors can be definitively subtyped in clinical settings, we also included breast cancers with unknown hormone receptor status as a separate category.

### Covariates and stratifying variables

Potential confounding variables were identified through a combination of literature review, directed acyclic graphs (DAGs), and stepwise regression analysis. The DAG that informed the selection of covariates in the model is shown in our previous publication [8]. All county-level covariate data were collected from the ACS 5-year estimates between 2017 and 2021 [20]. Covariates included in the multivariable model were recent poverty (the percentage of the county population living below the poverty line), the 2013 rural/urban continuum code, % Non-Hispanic Black, % Hispanic, % without health insurance, % with a bachelor’s degree or higher, and % unemployed. We did not adjust for individual behaviors (obesity, physical health, binge drinking, and recent smoking), because these factors were considered potential mediators on the causal pathway between persistent poverty and breast cancer incidence. Additionally, we did not adjust for the percentage of adults who received a mammogram in the previous two years, as this factor showed collinearity with the intercept in our linear regression model and is largely considered to be determined by socioeconomic factors included in the multivariable model.

County-level rural/urban status and the percentage of Non-Hispanic Black individuals in each county were used as stratifying variables in the analysis. Rural/urban status was classified according to the 2013 Rural Urban Continuum Codes (RUCC) developed by the United States Department of Agriculture (USDA) as follows: urban (RUCC codes 1–3) or rural (codes 4–9) [21]. After visually inspecting the distribution of the percentage of Non-Hispanic Black individuals across counties, we created a two-level indicator variable of counties with a Non-Hispanic Black population of ≤ 5% or > 5%.

### Statistical analysis

We accessed county-level age-adjusted breast cancer incidence rates between 2017 and 2021, 2013 RUCC codes, and 2015–2019 persistent poverty status using the SEER*Stat program [22]. We then merged these data with ACS 5-year estimates for each covariate by state and county name. We examined county characteristics according to persistent poverty status. We calculated mean age-adjusted incidence rates overall and for each breast cancer subtype and determined differences in incidence rates between persistently poor and non-persistently poor counties using t-tests.

Age-adjusted and multivariable linear regression models were conducted to examine the association between county-level persistent poverty and breast cancer incidence rates by breast cancer subtype. Results were reported as linear estimates of the rate difference (RD) between persistent poverty and non-persistent poverty counties. A linear regression model was selected given our focus on estimating differences in incidence rates, which are often more interpretable than relative measures. We assessed the linear regression model assumptions, confirming that the county-level incidence rates were normally distributed with relatively constant variance. We also stratified our results by rural/urban status and % Non-Hispanic Black. We determined statistical significance at the alpha < 0.05 level using 95% confidence intervals. Descriptive analysis and linear regression models were executed in SAS v9.4 [23]. The study was approved by the Michigan State University Institutional Review Board.

## Results

As shown in Table 1, most persistent poverty counties were rural (81.4%) and located in the South Census Region (88.5%). The percentages of Non-Hispanic Black (21.14% vs. 7.45%), Hispanic or Latino (18.20% vs. 14.60%), individuals living below the poverty line within the previous five years (25.99% vs. 14.32%), without health insurance (23.59% vs. 16.52%), and without employment (4.55% vs. 3.62%) were higher in persistent poverty compared to non-persistent poverty counties. A lower percentage of individuals in persistent poverty counties received a bachelor’s degree or higher compared to those in non-persistent poverty counties (14.41% vs. 21.84%) (Table 1).

**Table 1:** County-level characteristics by persistent poverty status from 2017 to 2021 (*n* = 1,079)

Characteristic	All counties (*n* = 1,079)	Persistent poverty (*n* = 156)	Non-persistent poverty (*n* = 923)
Rural/urban status, *n* (%)			
Rural	639 (59.2)	127 (81.4)	512 (55.5)
Urban	440 (40.8)	29 (19.6)	411 (44.5)
Census region, *n* (%)			
Northeast	105 (9.7)	1 (0.6)	104 (11.3)
South	592 (54.9)	138 (88.5)	454 (49.2)
Midwest	201 (18.6)	3 (1.9)	198 (21.5)
West	181 (16.8)	14 (9.0)	167 (18.1)
% Non-Hispanic Black, mean (SD)	9.42 (13.6)	21.14 (21.5)	7.45 (10.6)
% Hispanic or Latino, mean (SD)	15.12 (19.1)	18.20 (28.9)	14.60 (16.9)
% Current poverty, mean (SD)	16.01 (6.6)	25.99 (5.9)	14.32 (4.9)
% Without health insurance, mean (SD)	17.54 (8.7)	23.59 (9.7)	16.52 (8.1)
% Received Bachelor’s Degree or Higher, mean (SD)	20.77 (3.8)	14.41 (6.4)	21.84 (9.6)
% Unemployment, mean (SD)	3.76 (1.4)	4.55 (1.8)	3.62 (1.3)

**Fig. 1:**
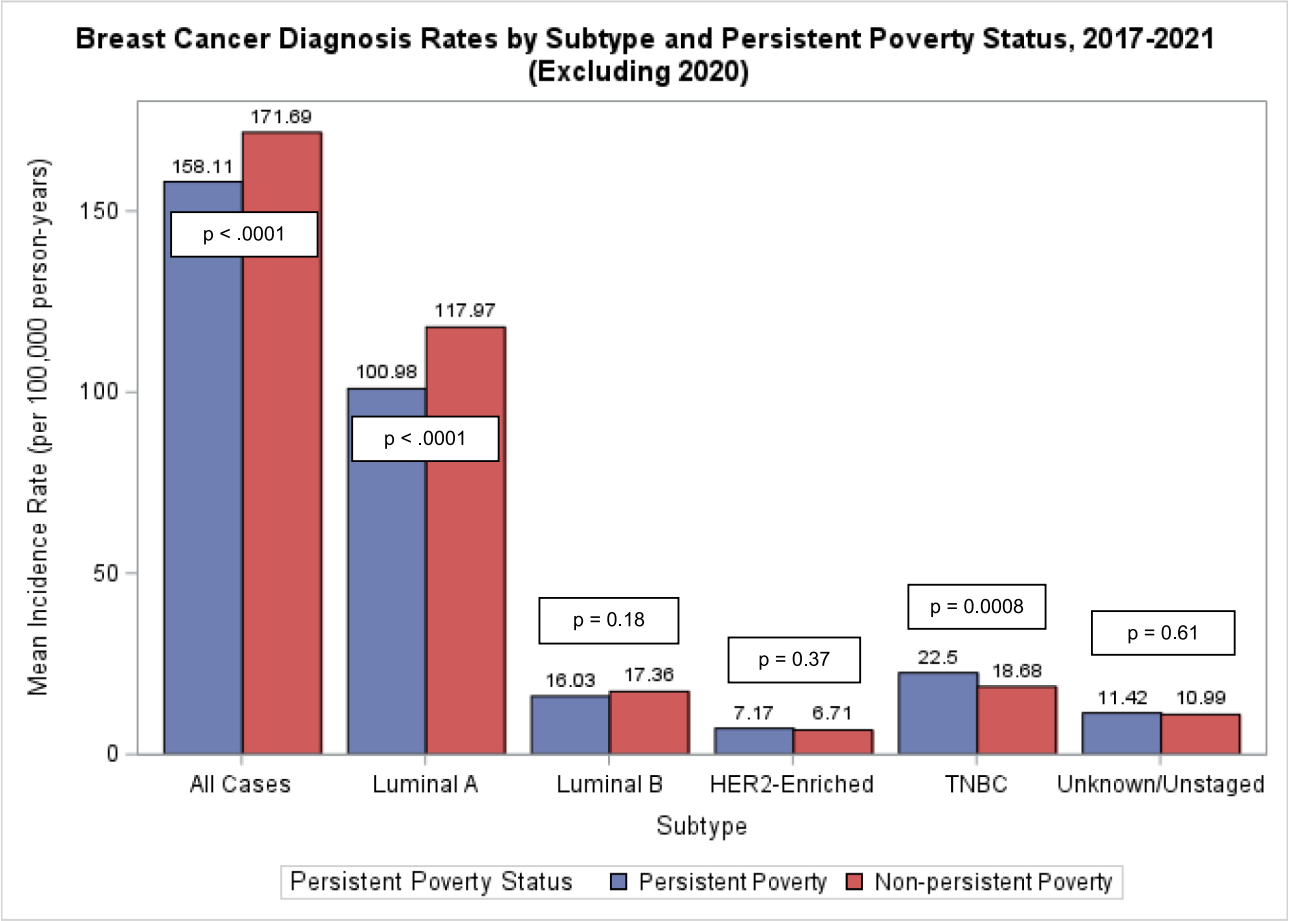
Mean age-adjusted breast cancer incidence rates within US counties by tumor subtype and county-level persistent poverty status from 2017 to 2021, excluding 2020 (*n* = 1,079)

Figure 1 shows the mean age-adjusted breast cancer incidence rates per 100,000 individuals overall and by tumor subtype within persistent poverty and non-persistent poverty counties. Counties not experiencing persistent poverty had higher age-adjusted rates of breast cancer overall (171.69 vs. 158.11 per 100,000), and Luminal A breast cancer (117.97 vs. 100.98 per 100,000) compared to persistent poverty counties. Higher rates of TNBC were observed in persistent poverty vs. non-persistent poverty counties (22.5 vs. 18.68 per 100,000)

Age-adjusted breast cancer incidence rates by subtypes across rurality and race strata are provided in Supplemental Tables 1 and 2. Overall, the highest age-adjusted breast cancer rates were evident in urban counties not experiencing persistent poverty (mean = 182.42/10,000 person-years, SD = 30.5), while the lowest rates were observed in rural persistent poverty counties (155.45/10,000 person-years, SD = 36.6). Age-adjusted Luminal A rates were lower, and TNBC rates were higher in persistent poverty vs. non-persistent poverty counties across both rural and urban county strata. In race-stratified analysis, age-adjusted breast cancer rates were highest in counties with larger Non-Hispanic Black populations not experiencing persistent poverty (175.91/10,000 person-years) and lowest in counties with smaller Non-Hispanic Black populations experiencing persistent poverty (153.94/10,000 person-years). Age-adjusted Luminal A rates were lower, and TNBC rates were higher in persistent poverty vs. non-persistent poverty counties across racial composition strata.

**Table 2:** Breast cancer incidence rate difference (RD) estimates (per 100,000 person-years) across subtypes between persistent poverty and non-persistent poverty counties in the US from 2017 to 2021 (excluding 2020)

	Number persistent poverty/ non-persistent poverty counties	Age-adjusted rate difference	Multivariable^a^ rate difference
All subtypes	156/923	− 13.58 (− 19.8, − 7.4)	2.36 (− 5.2, 9.9)
Luminal A		− 16.99 (− 22.1, − 11.9)	2.30 (− 3.7, 8.3)
Luminal B		− 1.33 (− 3.0, 0.4)	− 0.61 (− 2.9, 1.7)
HER2-enriched		0.46 (− 0.55, 1.5)	0.14 (− 1.2, 1.5)
Triple-negative		3.82 (2.0, 5.6)	1.12 (− 1.1, 3.4)
Unknown/unstaged		0.43 (− 1.3, 2.1)	− 0.59 (− 2.7, 1.5)

^a^Multivariable model adjusted for recent poverty, rural–urban continuum code, % without health insurance, % unemployed, % non-Hispanic Black, % Hispanic or Latino, and % with a bachelor’s degree or higher

As shown in Table 2, counties in persistent poverty had lower rates of age-adjusted breast cancer overall (RD = − 13.58, 95% CI = − 19.8, − 7.4) and for Luminal A breast cancer (RD = − 16.99, 95% CI = − 22.1, − 11.9), but higher rates of TNBC (RD = 3.82, 95% CI = 2.0, 5.6). Rate differences were not significant in the multivariable model. After adjusting for recent poverty, rural–urban continuum code, race and ethnicity, and other socioeconomic factors, Luminal A breast cancer rates were non-significantly higher in persistently poor counties (RD = 2.30, 95% CI = − 3.70, 8.31), while the higher TNBC incidence in persistently poor counties was attenuated (RD = 1.12, 95% CI = − 1.14, 3.38). Across subtypes, recent poverty was the primary factor influencing changes in rate differences between the age-adjusted and multivariable models.

**Fig. 2:**
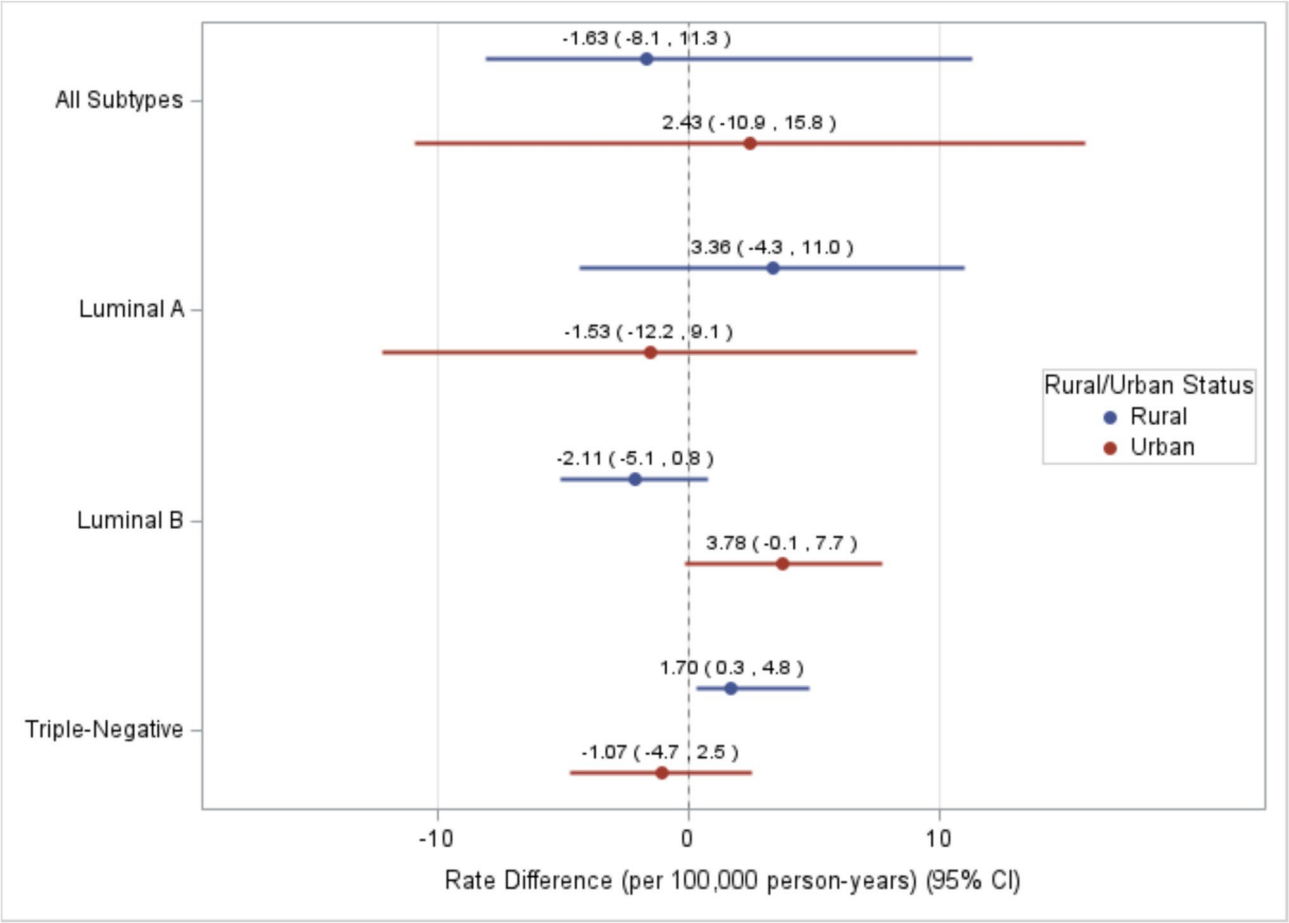
Multivariable breast cancer incidence rate difference (RD) estimates (per 100,000 person-years) across subtypes between persistent poverty and non-persistent poverty counties in the US from 2017 to 2021 (excluding 2020), stratified by rural/urban status (*n* = 1,079). Multivariable model adjusted for recent poverty, rural–urban continuum code, % without health insurance, % unemployed, % non-Hispanic Black, % Hispanic or Latino, and % with a bachelor’s degree or higher

**Fig. 3:**
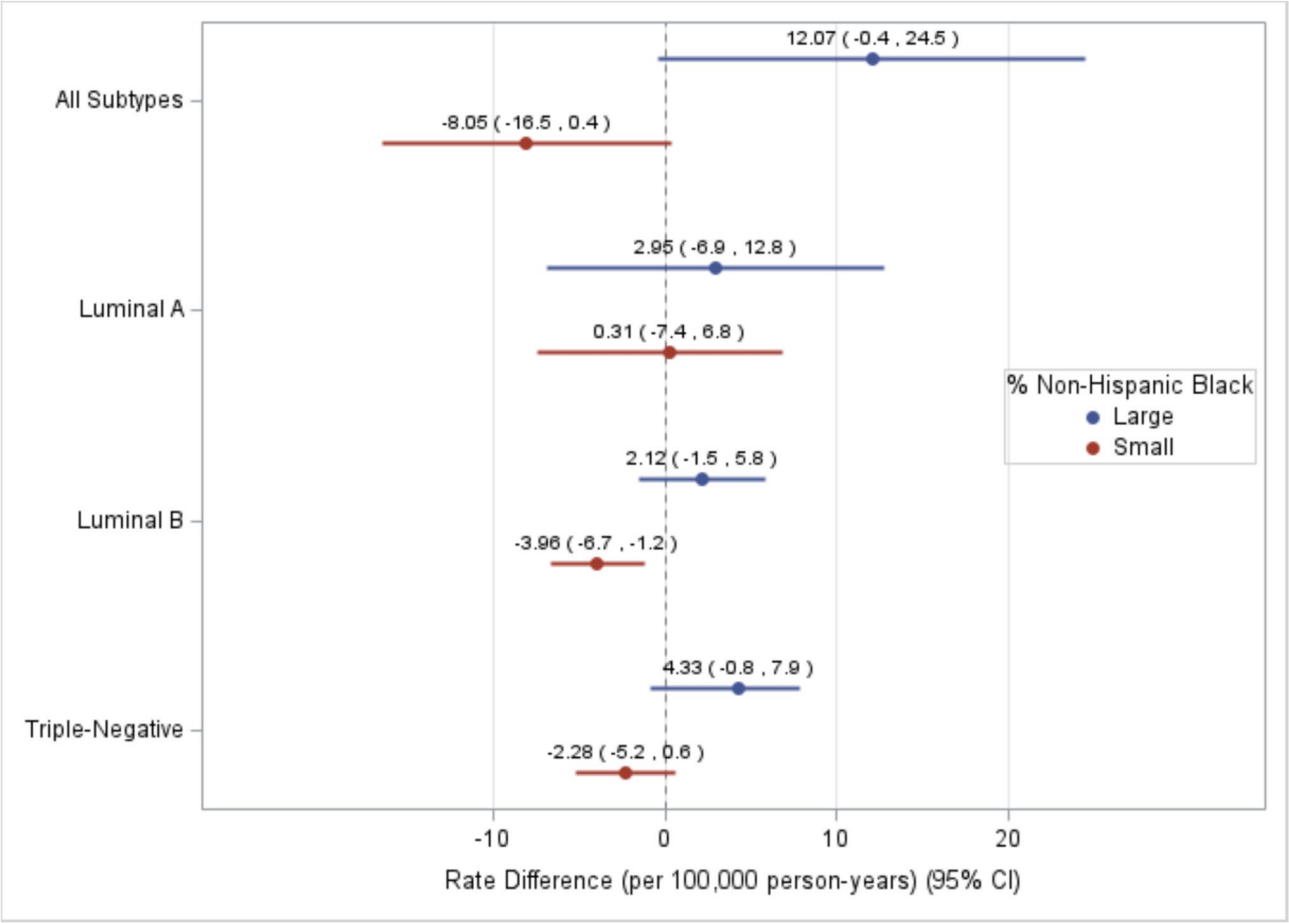
Multivariable breast cancer incidence rate difference (RD) estimates (per 100,000 person-years) across subtypes between persistent poverty and non-persistent poverty counties in the US from 2017 to 2021 (excluding 2020), stratified by % Non-Hispanic Black (*n* = 1,079). Multivariable model adjusted for recent poverty, rural-urban continuum code, % without health insurance, % unemployed, % Non-Hispanic Black, % Hispanic or Latino, and % with a bachelor’s degree or higher. % Non-Hispanic Black categorized into large (> 5%) and small (≤ 5%)

Results from the models stratified by county rural/urban status are shown in Fig. 2 and Supplemental Table 3. Rate differences in breast cancer subtypes by persistent poverty status did not significantly vary according to rural–urban status in multivariable models (all *p*_int_ ≥ 0.09). However, higher TNBC rates were observed in persistently poor rural (RD = 1.70, 95% CI = 0.3, 4.8) but not urban (RD = − 1.07, 95% CI = − 4.7, 2.5; *p*_int_ = 0.09) counties. In race-stratified analysis, we observed significant interaction for Luminal B (*p*_int_ = 0.03) and unknown (*p*_int_ = 0.01) subtypes (Supplemental Table 4). Specifically, Luminal B rates were lower in persistent poverty vs. non-persistent poverty counties with a larger NHB population after adjusting for covariates (RD = − 3.96, 95% CI = − 6.7, − 1.2), with no significant difference in counties with a smaller NHB population (RD = 2.12, 95% CI = − 1.5, 5.8) (Fig. 3).

## Discussion

### Role of persistent vs. recent poverty

Findings from this study suggest that persistent poverty is not independently associated with breast cancer incidence by subtype. While age-adjusted breast cancer subtype incidence rates differed by persistent poverty, these differences were not apparent after adjusting for current sociodemographic factors. However, persistent poverty may contribute to higher TNBC rates in rural areas and lower Luminal B rates in counties with larger non-Hispanic Black residents. Results from this study add to our understanding of the role of persistent poverty in breast cancer subtype disparities and suggest that differences are largely explained by other measures of recent disadvantage, including current poverty, unemployment, uninsurance, and race.

### Findings and implications

To our knowledge, this study was the first to assess differences in breast tumor subtype incidence rates by persistent poverty status, although previous studies have assessed differences by other measures of disadvantage. Our finding of lower age-adjusted Luminal A breast cancer incidence rates in persistent poverty counties is similar to results of a previous study where a lower proportion of HR+ to HR− subtypes was observed in socioeconomically disadvantaged neighborhoods [24]. Lower Luminal A breast cancer rates in persistent poverty counties may reflect limited access to screening mammography, which could lead to fewer diagnoses of early-stage, HR+cancers [25]. The higher rates of TNBC in persistent poverty counties observed in this study are also consistent with results from a study demonstrating associations between census tract-level persistent poverty and TNBC, as well as high-grade and late-stage tumors [26]. Breast cancer subtype differences by persistent poverty may reflect variation in reproductive factors associated with breast cancer, such as breastfeeding, age at first birth, and parity [27]. Importantly, our findings suggest that these differences are largely explained by other measures of disadvantage including recent poverty. From a public health perspective, these findings suggest that addressing recent poverty may be an effective strategy to mitigate breast cancer disparities, even in communities with a history of long-standing structural disadvantage. Nonetheless, these factors do not completely explain differences in breast cancer subtype rates according to persistent poverty status in rural counties and those with larger non-Hispanic Black residents. Thus, broader structural reforms to address racial segregation, rural workforce shortages, and long-term economic marginalization in these communities are needed.

### Geographic and racial differences

This study was the first to our knowledge to assess whether differences in breast cancer subtypes by persistent poverty status varied according to rural/urban status and race. Previous research highlights the higher proportion of TNBC in rural areas and among impoverished populations, [28]. In this study, we observed larger differences in TNBC rates by persistent poverty status in rural counties, though no significant heterogeneity by rural/urban status was evident in multivariable models. Conversely, the difference in Luminal A breast cancer rates in persistent poverty compared to non-persistent poverty counties was larger in urban vs. rural counties. These findings suggest that the intersection of geography and short- and long-term economic disadvantage may differentially influence breast cancer diagnosis by tumor subtype. Elevated TNBC rates in rural and persistently poor counties are concerning as TNBC is more difficult to treat, and these areas face limited healthcare access [29], as well as greater incidence of late-stage breast cancer diagnosis [8]. Addressing these disparities will require geographically tailored solutions, such as improving healthcare delivery infrastructure and targeting screening and early-detection resources in rural regions. Specifically, expanding breast cancer screening in community health centers, implementing Medicaid expansion, and increasing incentives for providers in health workforce shortage areas may improve early detection of breast cancer, including TNBC. However, due to the ecological nature of our study, individual-level research is needed to guide specific policy recommendations aimed at reducing disparities.

Given well-recognized racial disparities in TNBC incidence, we specifically stratified our analysis by the percentage of Non-Hispanic Black individuals within each county to further inform our understanding of potential drivers of these disparities. Consistent with previous studies [30–32], we observed the highest rates of TNBC in persistently poor counties with larger Non-Hispanic Black populations. Interestingly, after adjusting for measures of recent disadvantage, higher rates of TNBC in persistently poor counties were only evident in counties with a smaller Non-Hispanic Black population. These findings, though not significant, suggest that persistent poverty may play a greater role in areas with fewer racial minorities, whereas recent disadvantage is more strongly accounting for higher TNBC rates in counties with a larger NHB population. Potential explanations for the lower rates of Luminal B in persistent poverty counties with larger NHB populations and higher rates of TNBC in persistent poverty counties with smaller NHB populations, even after adjusting for recent measures of disadvantage, are unclear. Although lower rates of protective factors like breastfeeding, and earlier, more frequent childbearing without lactation have been linked to increased TNBC risk, particularly among NHB women [33, 34], these patterns do not fully explain why persistent poverty was associated with higher TNBC rates only in counties with smaller NHB populations. Further investigation is needed to understand these associations. To mitigate long-standing breast cancer inequities, research to identify and address the specific drivers of place-based inequities, such as limited infrastructure, healthcare barriers, and other factors that may transcend race, is urgently needed.

### Strengths and limitations

This study has several strengths. First, we used the most recent SEER population-based cancer registry data, including all breast cancer cases, and publicly available ACS data, both regarded as high-quality and generalizable to the U.S. population. Additionally, we used robust multivariable regression models to evaluate the independent association of persistent poverty on breast cancer subtypes, a novel contribution to the recent literature. Our study does have limitations, including reliance on dichotomous categories to assess variation in associations by rural/urban status and race. This approach does not precisely capture the potential impact of these factors on the observed differences in breast cancer subtypes by persistent poverty. Moreover, our focus on rural–urban differences meant that some SEER incidence rates were derived from small case counts, reducing statistical reliability and increasing estimate variability. Although the SEER registry program has data suppression rules in place to prevent the misinterpretation of unstable incidence rates that result from small case counts. Finally, ecological studies have inherent limitations, including the potential for the ecological fallacy, through which group-level characteristics are attributed to individuals; in our study, group-level characteristics included county-level persistent poverty status. Residual confounding by other factors, including unmeasured health behaviors and individual-level risk factors, is possible, warranting future analysis of associations between persistent poverty and breast cancer subtypes at the individual level.

## Conclusion

Our findings highlight the confounding role of recent poverty and other sociodemographic factors on the relationship between persistent poverty and incidence of breast cancer subtypes. However, persistent poverty may independently contribute to higher TNBC rates in rural areas and lower Luminal B rates in counties with larger non-Hispanic Black residents. These findings underscore the need for targeted prevention and early-detection strategies to address breast cancer disparities within socioeconomically disadvantaged communities.

## Supplementary Information

Below is the link to the electronic supplementary material.

Supplementary file1 (DOCX 249 kb)

## Data Availability

No datasets were generated or analyzed during the current study.
