# Supplementary material for: Persistent poverty and breast cancer incidence by tumor subtype: intersections of rural/urban residence and race within USA Surveillance Epidemiology and End Results Registries, 2017 to 2021
Source: Cancer Causes Control. 2026 Jan 17;37(2):26. doi: 10.1007/s10552-025-02114-z (PMC12812092; doi:10.1007/s10552-025-02114-z)
Supplement: Supplementary file 1 — Supplementary file1 (DOCX 249 kb) [file 10552_2025_2114_MOESM1_ESM.docx]

**Supplemental Figure 1: Directed Acyclic Graph (DAG) of persistent poverty, late-stage breast cancer, and included covariates.**

**
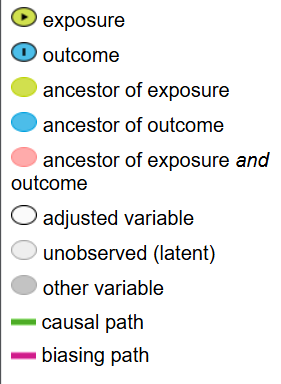

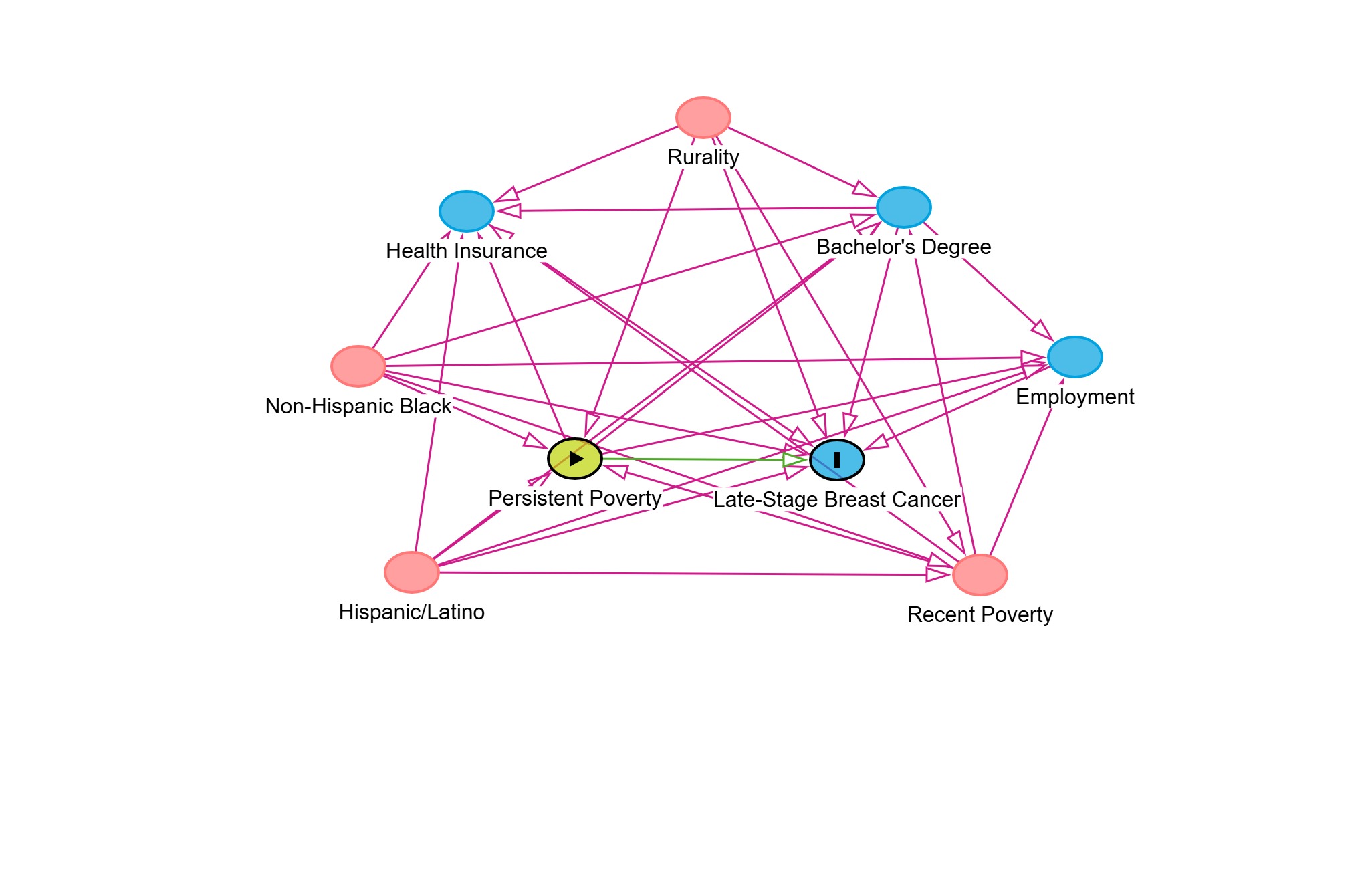
**

Adapted from “Persistent poverty and late-stage breast cancer diagnosis in the United States: Impacts of rural residence, race, and time within Surveillance Epidemiology and End Results registries, 2004 to 2021” by Sherr et al., 2025.^8^

**Supplemental Table 1: Mean age-adjusted breast cancer incidence rates (per 100,000 person-years) across subtypes between persistent poverty and non-persistent poverty counties in the US from 2017-2021 (excluding 2020), stratified by rural/urban status.**

|  | Rural Counties (n = 639) | | Urban Counties (n = 440) | |
| --- | --- | --- | --- | --- |
|  | Persistent Poverty Counties (n = 127) | Non-persistent Poverty Counties (n = 512) | Persistent Poverty Counties (n = 29) | Non-persistent Poverty Counties (n = 411) |
|  | Mean Breast Cancer Incidence Rate (SD) | Mean Breast Cancer Incidence Rate (SD) | Mean Breast Cancer Incidence Rate (SD) | Mean Breast Cancer Incidence Rate (SD) |
| All Subtypes | 155.45 (36.6) | 163.09 (37.6) | 169.75 (45.1) | 182.42 (30.5) |
| Luminal A | 99.87 (27.9) | 110.93 (31.2) | 105.83 (22.3) | 126.72 (26.8) |
| Luminal B | 15.16 (11.0) | 17.11 (10.6) | 19.86 (12.9) | 17.67 (8.5) |
| HER2-Enriched | 7.01 (5.9) | 6.38 (7.0) | 7.90 (4.5) | 7.13 (4.5) |
| Triple-Negative | 22.42 (13.0) | 17.55 (11.2) | 22.83 (15.4) | 20.07 (8.0) |
| Unknown/Unstaged | 10.99 (9.8) | 11.12 (11.0) | 13.33 (14.8) | 10.83 (7.9) |

**Supplemental Table 2: Mean age-adjusted breast cancer incidence rates (per 100,000 person-years) across subtypes between persistent poverty and non-persistent poverty counties in the US from 2017-2021 (excluding 2020), stratified by % Non-Hispanic Black.**

|  | **> 5% Non-Hispanic Black (n = 443)** | | **≤ 5% Non-Hispanic Black (n = 636)** | |
| --- | --- | --- | --- | --- |
|  | **Persistent Poverty Counties (n = 85)** | **Non-persistent Poverty Counties (n = 358)** | **Persistent Poverty Counties (n = 71)** | **Non-persistent Poverty Counties (n = 565)** |
|  | **Mean Breast Cancer Incidence Rate (SD)** | **Mean Breast Cancer Incidence Rate (SD)** | **Mean Breast Cancer Incidence Rate (SD)** | **Mean Breast Cancer Incidence Rate (SD)** |
| **All Subtypes** | **161.59 (35.3)** | **175.91 (27.0)** | **153.94 (42.1)** | **169.02 (40.4)** |
| **Luminal A** | **104.05 (25.6)** | **117.29 (24.3)** | **97.30 (28.4)** | **118.40 (33.6)** |
| **Luminal B** | **15.22 (10.2)** | **17.77 (8.2)** | **17.00 (12.9)** | **17.09 (10.6)** |
| **HER2-Enriched** | **7.50 (6.1)** | **7.22 (4.4)** | **6.78 (5.2)** | **6.39 (6.9)** |
| **Triple-Negative** | **25.15 (13.2)** | **21.56 (8.64)** | **19.32 (13.1)** | **16.85 (10.3)** |
| **Unknown/Unstaged** | **9.66 (7.6)** | **12.08 (8.4)** | **13.53 (13.7)** | **10.29 (10.4)** |

**Supplemental Table 3: Age-adjusted and multivariable breast cancer incidence rate difference (RD) estimates (per 100,000 person-years) across subtypes between persistent poverty and non-persistent poverty counties in the US from 2017-2021 (excluding 2020), stratified by rural/urban status.**

|  | **Rural Counties (n = 639)** | | | **Urban Counties (n = 440)** | | |  |  |
| --- | --- | --- | --- | --- | --- | --- | --- | --- |
|  | **Number Persistent Poverty/ Non-persistent Poverty Counties** | **Age-adjusted Rate Difference** | **Multivariable^a^ Rate Difference** | **Number Persistent Poverty/ Non-persistent Poverty Counties** | **Age-adjusted Rate Difference** | **Multivariable^a^ Rate Difference** | **Age-adjusted p_int_^b^** | **Multivariable p_int_^c^** |
| All Subtypes | 127/512 | -7.63 (-14.9, -0.4) | 1.63 (-8.1, 11.33) | 29/411 | -12.66 (-24.6, -0.7) | 2.43 (-10.9, 15.8) | <.0001 | 0.61 |
| Luminal A |  | -11.05 (-1.0, -5.1) | 3.36 (-4.3, 11.0) |  | -20.89 (-30.9, -10.9) | -1.53 (-12.2, 9.1) | <.0001 | 0.30 |
| Luminal B |  | -1.95 (-4.0, 0.1) | -2.11 (-5.1, 0.8) |  | 2.19 (-1.2, 5.5) | 3.78 (-0.1, 7.7) | 0.04 | 0.09 |
| HER2-Enriched |  | 0.63 (-0.7, 2.0) | 0.68 (-1.8, 1.9) |  | 0.77 (-0.9, 2.5) | 0.49 (-1.5, 2.5) | 0.18 | 0.96 |
| Triple-Negative |  | 4.87 (2.6, 7.1) | 1.70 (0.3, 4.8) |  | 2.76 (-0.5, 6.0) | -1.07 (-4.7, 2.5) | <.0001 | 0.09 |
| Unknown/Unstaged |  | -0.13 (-2.2, 2.0) | -1.53 (-4.3, 1.3) |  | 2.50 (-0.7, 5.7) | 0.75 (-2.8, 4.3) | 0.62 | 0.53 |

^a^ Multivariable model adjusted for age, recent poverty, rural-urban continuum code, % without health insurance, % unemployed, % Non-Hispanic Black, % Hispanic or Latino, and % with a bachelor’s degree or higher.

^b^ p-value for interaction based on age-adjusted model.

^c^ p-value for interaction based on multivariable model.

**Supplemental Table 4: Age-adjusted and multivariable breast cancer incidence rate difference (RD) estimates (per 100,000 person-years) across subtypes between persistent poverty and non-persistent poverty counties in the US from 2017-2021 (excluding 2020), stratified by % Non-Hispanic Black.**

|  | **> 5% Non-Hispanic Black (n = 443)** | | | **≤ 5% Non-Hispanic Black (n = 636)** | | |  |  |
| --- | --- | --- | --- | --- | --- | --- | --- | --- |
|  | Number Persistent Poverty/ Non-persistent Poverty Counties | **Age-adjusted Rate Difference** | **Multivariable^a^ Rate Difference** | **Number Persistent Poverty/ Non-persistent Poverty Counties** | **Age-adjusted Rate Difference** | **Multivariable^a^ Rate Difference** | **Age-adjusted p_int_^b^** | **Multivariable p_int_^c^** |
| All Subtypes | 85/358 | -14.32 (-21.1, -7.5) | -8.05 (-16.5, 0.4) | 71/565 | -15.08 (-25.1, -5.1) | 12.07 (-0.4, 24.5) | <.0001 | 0.37 |
| Luminal A |  | -13.23 (-19.1, -7.4) | 0.31 (-7.4, 6.8) |  | -21.10 (-29.3, -12.9) | 2.95 (-6.9, 12.8) | <.0001 | 0.39 |
| Luminal B |  | -2.55 (-4.6, -0.5) | -3.96 (-6.7, -1.2) |  | -0.09 (-2.8, 2.6) | 2.12 (-1.5, 5.8) | 0.03 | 0.03 |
| HER2-Enriched |  | 0.28 (-0.8, 1.4) | -1.00 (-2.5, 0.5) |  | 0.39 (-1.3, 2.0) | 1.79 (-0.5, 4.0) | 0.11 | 0.65 |
| Triple-Negative |  | 3.59 (1.3, 5.9) | -2.28 (-5.2, 0.6) |  | 2.48 (-0.2, 5.1) | 4.33 (-0.8, 7.9) | <.0001 | 0.28 |
| Unknown/Unstaged |  | -2.42 (-4.4, -0.5) | -0.49 (-3.0, 2.0) |  | 3.23 (-0.6, 5.9) | 0.88 (-2.5, 4.3) | 0.98 | 0.01 |

^a^ Multivariable model adjusted for age, recent poverty, rural-urban continuum code, % without health insurance, % unemployed, % Non-Hispanic Black, % Hispanic or Latino, and % with a bachelor’s degree or higher.

^b^ p-value for interaction based on age-adjusted model.

^c^ p-value for interaction based on multivariable model.
